# *Ex vivo* radiation sensitivity assessment for individual head and neck cancer patients using deep learning-based automated nuclei and DNA damage foci detection

**DOI:** 10.1016/j.ctro.2024.100735

**Published:** 2024-01-30

**Authors:** I. Lauwers, K.S. Pachler, M.E. Capala, N.D. Sijtsema, D.C. Van Gent, M. Rovituso, M.S. Hoogeman, G.M. Verduijn, S.F. Petit

**Affiliations:** a Department of Radiotherapy, Erasmus MC Cancer Institute, University Medical Center Rotterdam, Rotterdam, the Netherlands; b Department of Molecular Genetics, Erasmus MC, University Medical Center Rotterdam, Rotterdam, the Netherlands; c Department of Radiology and Nuclear Medicine, Erasmus MC, University Medical Center Rotterdam, Rotterdam, the Netherlands; d Holland Proton Therapy Center, Delft, the Netherlands; e Department of Medical Physics and Informatics, HollandPTC, Delft, the Netherlands

## Abstract

**Introduction:**

Tumor biopsy tissue response to *ex vivo* irradiation is potentially an interesting biomarker for *in vivo* tumor response, therefore, for treatment personalization. Tumor response *ex vivo* can be characterized by DNA damage response, expressed by the large-scale presence of DNA damage foci in tumor nuclei. Currently, characterizing tumor nuclei and DNA damage foci is a manual process that takes hours per patient and is subjective to inter-observer variability, which is not feasible in for clinical decision making. Therefore, our goal was to develop a method to automatically segment nuclei and DNA damage foci in tumor tissue samples treated with radiation *ex vivo* to characterize the DNA damage response, as potential biomarker for *in vivo* radio-sensitivity.

**Methods:**

Oral cavity tumor tissue of 21 patients was irradiated *ex vivo* (5 or 0 Gy), fixated 2 h post-radiation, and used to develop our method for automated nuclei and 53BP1 foci segmentation. The segmentation model used both deep learning and conventional image-analysis techniques. The training (22 %), validation (22 %), and test set (56 %) consisted of thousands of manually segmented nuclei and foci. The segmentations and number of foci per nucleus in the test set were compared to their ground truths.

**Results:**

The automatic nuclei and foci segmentations were highly accurate (Dice = 0.901 and Dice = 0.749, respectively). An excellent correlation (R^2^ = 0.802) was observed for the foci per nucleus that outperformed reported inter-observation variation. The analysis took ∼ 8 s per image.

**Conclusion:**

This model can replace manual foci analysis for *ex vivo* irradiation of head-and-neck squamous cell carcinoma tissue, reduces the image-analysis time from hours to minutes, avoids the problem of inter-observer variability, enables assessment of multiple images or conditions, and provides additional information about the foci size. Thereby, it allows for reliable and rapid *ex vivo* radio-sensitivity assessment, as potential biomarker for response *in vivo* and treatment personalization.

## Highlights

• The model assesses patient-specific radiosensitivity in *ex vivo* HNSCC tissue.
• The model is based on 53BP1 DNA damage foci and nuclei microscopy images.
• The model is based on a deep learning and conventional image analysis techniques.
• This model can replace manual foci analysis for *ex vivo* HNSCC tissue.
• The model reduces the image-analysis time and avoids inter-observer variability.

## 1 Introduction

The five-year overall survival rate of head and neck squamous cell carcinoma (HNSCC) remains low at approximately 60–70 %, while many patients experience long-lasting side effects [1], [2], [3]. HNSCC is a complex and heterogeneous disease, often associated with tobacco and/or alcohol consumption, or human papillomavirus (HPV) infection [4], [5], [6]. Therefore, personalized treatment could be beneficial for this patient group, but there are currently no clinically validated biomarkers available to predict the individual patient response, required to stratify patients for personalized treatments [7], [8], [9], [10], [11], [12]. A potential biomarker for response prediction is the *ex vivo* response of tumor tissue to radiation treatment, which has been shown to correlate to *in vivo* response [13]. A recent study demonstrated that HNSCC tumor tissue from patients can remain viable *ex vivo* for five days, allowing for *ex vivo* treatment and response assessment that would fit within the timeframe of clinical decision-making [14].

*Ex vivo* radio-sensitivity can be quantified by studying DNA damage foci, which are accumulations of DNA damage repair proteins that appear near double strand DNA breaks. The number of foci per nucleus closely correlates to the number of double strand breaks [15], [16], [17]. Moreover, DNA damage focus’ properties *e.g.* size can provide additional information about the complexity of the DNA damage [18], [19].

Manual analysis of foci and nuclei images generated during *ex vivo* radio-sensitivity experiments with tumor tissue (*e.g.* biopsies) is currently not feasible because it can take hours per image and is prone to inter-observer variation. Even experts were recorded to count significantly different numbers of foci [20]. Moreover, large intra-sample differences can be found within one sample, thus requiring assessment of multiple images per sample and condition, further increasing the analysis time [21]. Automated counting and segmentation tools have been developed for *in vitro* cell cultures [20], [22], [23], but are not suitable for samples with extreme differences in foci number and properties, background signal, and cell clustering, which are all often observed in tissue samples [24], [25]. Currently, the lack of an accurate method for counting nuclei and foci in tumor tissue samples is an important bottleneck for *ex vivo* radiation sensitivity studies.

This study aimed to develop an automatic segmentation model that can accurately segment nuclei and DNA damage foci in tumor tissue samples treated with *ex vivo* radiation. Resection material of HNSCC tumors was treated with either X-ray or proton irradiation or left untreated. DAPI and 53BP1 were used to stain nuclei and DNA damage foci, respectively. After segmentation, the number of foci and their size were automatically counted. Next, the accuracy and applicability of this model was tested by first assessing the segmentation accuracy, secondly, by determining whether the model could be used on samples treated with different treatment modalities (photons and protons), and thirdly, by assessing the intra sample variation between multiple images in the same condition.

## 2 Methods

### 2.1 Patients, samples and *ex vivo* treatment

Fresh tumor tissue was obtained from primary tumors of 21 oral cavity SCC (OCSCC) patients who underwent surgical resection at the Erasmus University Medical Center (Erasmus MC), The Netherlands in accordance with the code of proper secondary use of human tissue established by the Dutch Federation of Medical Scientific Societies, and approved by the Erasmus MC Medical Ethical Committee (MEC-2017–1049). The tissue was prepared, sliced, and cultured as described by Capala *et al*. (2023) [14]. For each patient, one tumor slice was kept untreated, and one was treated with photons (single dose of 5 Gy) using an X-Strahl RS320 X-ray cabinetwith 0.5 mm Cu filter at 195 keV. A multiple well plate (six wells: 85.2x127.8 mm^2^) containing tissue sample was placed in the middle of the radiation beam with a diameter of 221.4 mm and was irradiated with a dose rate of 1.6 Gy/min.

For three patients, an additional slice was treated with proton irradiation (single dose of 5 Gy, not RBE corrected). The proton irradiations were all performed at HollandPTC in the R&D beam line. From a therapeutic pencil beam, a passive scattering system produced a field size of a 10x10 cm^2^, with dose uniformity of 98 % ±2% over the whole area, irradiating the whole sample uniformly with a dose rate of 1.5 Gy/min. To irradiate the sample evenly over its depth (beam direction), a 3D range energy modulator was used, with an initial energy of 150 MeV, creating a Spread-out-Bragg peak (SOBP) of 25 mm with 98 % ±1% uniformity. A multiple well plate (four wells: 85.2x85.2 mm^2^) containing tissue sample was then placed at the middle of the SOBP, using a solid plastic phantom of RW3 material [26].

### 2.2 Staining, microscopy and slice selection

After irradiation, the slices were cultured for two hours, after which they were fixed, prepared for immunostaining, and stained with DAPI and 53BP1. Culture conditions and immunostaining procedures were described previously [14]. Per condition, for each slice ten images were acquired at different locations under the Leica Stellaris Incidence Angle microscope with a 63x objective. Detailed Z-stacks were acquired that captured the entire thickness of the slice with a step size of 1 µm. The images were 183.65x183.65 µm^2^ (1024x1024 pixels). Using the 405 and 488 iodine lasers, the nuclei and 53BP1 foci were imaged in blue and green respectively. Despite different labeling efficiencies, imaging settings were kept constant per channel for two reasons. First, to obtain nuclei images with variable quality, ensuring that the model was also robust for low quality images. Second, to enable the comparison of foci counts between images.

In contrast to cell cultures, individual cells often lay on top of each other in tissue cultures, making maximum projections suboptimal. To ensure individual nuclei distinction, while still including a large number of nuclei, the slice with the highest average intensity in the DAPI-channel Z-stack was selected for further processing. For 53BP1 foci, a maximum projection of three slices around the selected DAPI slice was used, ensuring that foci that were slightly out of focus could be distinguished from background noise. Slice selection was performed automatically using Python 3.8.

### 2.3 The ground truth nuclei and foci segmentations

The nuclei ground truths were manually annotated using the open-source software APEER in the DAPI channel. The ground truths of the foci channel were derived semi-automatically in open source software ImageJ using a Random Forest algorithm. The algorithm performed well when trained on one individual image, but it was not generalizable to other images without retraining and manual inspection. Therefore, the model was retrained manually for each image (Supplementary Information 1). During manual and semi-automatic annotating, contrast was adapted dynamically to ensure all the nuclei and foci were differentiable from background staining. All annotations were made by a single observer to avoid inter-observer variability.

### 2.4 The training, validation and test set

The patients were split into a training, validation, and test set with five, five and eleven patients respectively. For each patient, one image of an untreated sample, one of a sample treated with photons and, if available, one of a sample treated with protons was used, resulting in ten, ten, and twenty-five images in the training, validation and test set respectively (Table 1). Each image contained hundreds of nuclei and even more foci, so the method was trained, validated and tested on thousands of nuclei and foci.

**Table 1:** Overview of patients and conditions in training, validation, and test set.

	Number of patients	Number of patients per condition (one image per patient per condition)			Total number of images
		Control (0 Gy)	Photons (5 Gy)	Protons (5 Gy)	
Training set	5 (24 %)	5	5	0	10 (22 %)
Validation set	5 (24 %)	5	5	0	10 (22 %)
Test set	11 (52 %)	11	11	3	25 (56 %)

### 2.5 Automated nuclei segmentation

Nuclei segmentation consisted of a combination of deep learning and conventional image analysis techniques. First, semantic segmentation took place using a deep learning model, which means every pixel in the images was classified as either a nucleus pixel or a background pixel. Second, conventional image analysis post-processing steps were performed to improve segmentations, segment individual nuclei, and filter out partial nuclei and background noise, and subsequently count the total number of nuclei.

#### 2.5.1 Semantic segmentation of nuclei

Nuclei were segmented using a U-net model [27]. The standard model architecture was expanded with image normalization before the first layer and batch normalization after each layer. Moreover, all images were down sampled to 512x512.

During model training, data augmentation was applied in the form of eight flips and rotations and by adapting the contrast with a factor 0.8 and 1.2. The model was trained with a batch size of 32 for 200 epochs. The Jaccard index loss function was minimized using an Adam optimizer with learning rate 10–3. The weights resulting in the lowest loss value for the validation set were used for further analysis. Semantic segmentation was implemented using the TensorFlow Keras toolbox and run on two CPUs(: Intel(R) Xeon(R) Gold 6248 CPU @ 2.50 GHz (20 CPU cores per processor); RAM: 755 GB) in Python 3.8.

#### 2.5.2 Post-processing of the nuclei segmentations

The U-net segmentations were post processed automatically using the open source software ImageJ (FIJI). First, holes in the middle of nuclei were automatically closed using hole filling and clustered nuclei were automatically separated using a watershed operation (FIJI setting tolerance 4). Afterwards, segmentations crossing the image boundary and segmentations smaller than 10 µm^2^ were excluded.

### 2.6 Automated foci segmentation

The same U-net structure, data augmentation, and training characteristics were used for the foci segmentation as for the nuclei model, only down sampling was omitted to maintain fine details necessary to accurately segment the foci. Because larger images needed more memory, the batch size was decreased from 32 to eight. The semantic foci segmentations were used without post processing.

### 2.7 Combining the nuclei and foci segmentations

**Fig. 1:**
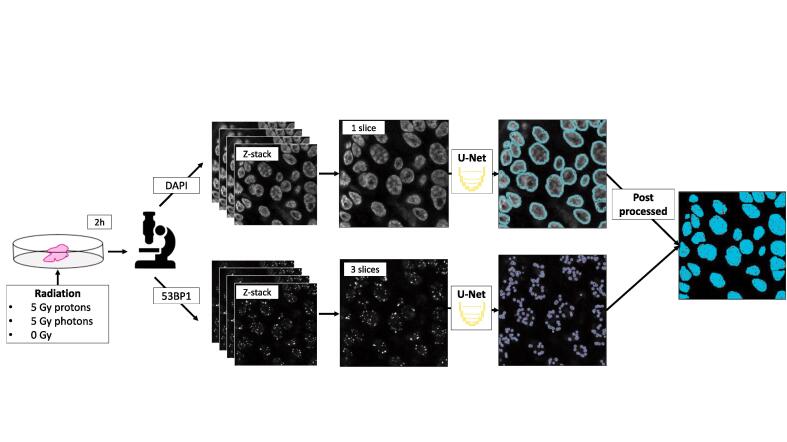
A schematic overview of the segmentation pipeline. OPSCC tissue was irradiated with either protons or photon ex vivo, or not irradiated. The DNA damage two hours after irradiation was visualized with a confocal microscope using a DAPI and 53BP1 dye for the nuclei and foci, respectively. In the DAPI channel, the image of the Z-stack with the highest average intensity was selected. The corresponding image in the foci Z-stack was selected and combined with the two adjacent images into a maximum projection. A U-net was used to make semantic segmentations for both the foci and nuclei images. The nuclei images were subsequently post processed and combined with the foci images. The output of the pipeline are the number of nuclei per image, the number of foci, and the number of foci per nucleus.

The foci and nuclei segmentations were overlaid, thus only including the foci inside nuclei segmentations. This filtered out background noise and enabled the calculation of the number foci per nuclear volume. For an overview of the total segmentation pipeline, see Fig. 1.

### 2.8 Performance evaluation

The performance of the U-net models was assessed with the pixelwise Dice similarity coefficient (DSC_pixel_) and Intersection over Union (IoU_pixel_) (Equations (1), (2)) in the test cohort. For the DSC_pixel_ and IoU_pixel_, the number of true positives (TP) was defined as the number of pixels that was correctly identified as foci/nuclei and the sum of the false positives (FP) and the false negatives (FN) was defined as the number of pixels that was incorrectly identified as foci/nuclei or background. Also the objectwise DSC (DSC_object_) was assessed for the foci U-net (Equation (1)). For the DSC_object_, the TP and the sum of the FP and NF were defined as the correctly and incorrectly identified foci respectively. Foci were deemed correctly identified if the foci centers in the prediction and ground truth were less than three pixels (the average foci size (2.53 pixels) rounded up) apart.

(1) $$\mathrm{DSC}=\frac{2|prediction\cap groundtruth|}{\left|prediction|+\right|\mathrm{groundtruth}|}=\frac{2TP}{2TP+FN+FP}$$

(2) $$\mathrm{IoU}=\frac{\left|prediction\cap groundtruth\right|}{\left|prediction|+\right|\mathrm{groundtruth}|-|prediction\cap groundtruth|}=\frac{\mathrm{TP}}{\mathrm{TP}+FN+FP}$$

For the foci segmentation the performance of the models in different conditions was evaluated. DSC_pixel_ were compared between treated versus untreated samples (paired T-test, n = 11) and between samples treated with protons versus photons (no test, n = 3).

The total model prediction depended on both the nuclei and foci segmentation. The total model‘s performance was analyzed by the Pearson correlation coefficient between the predictions and ground truths. The foci count per nuclear volume was calculated by dividing the number of foci in a nucleus by the area of the nucleus in the selected Z-plan and the height of the maximum projection.

### 2.9 Applying the model to compare DNA damage in different conditions

After the model was trained and validated, it was used to determine differences in DNA damage after proton, photon and no irradiation and to assess intra-tumor intra-condition differences using the ten images per condition (proton, photon and no irradiation), for the patients in the test cohort. The number of foci per nuclear volume and size were compared per patient between photon and control conditions using a Kruskal Wallis test. When comparing three groups (control, photon and proton), a Kruskal Wallis test was followed by a Dunn’s post hoc test if the Kruskal Wallis test was significant. A p-value < 0.05 was considered statistically significant. The intra-tumor intra-condition variation was presented using descriptive statistics and expressed as the standard deviation between the different images belonging to the same sample and condition relative to the average of that sample and condition.

## 3 Results

**Fig. 2:**
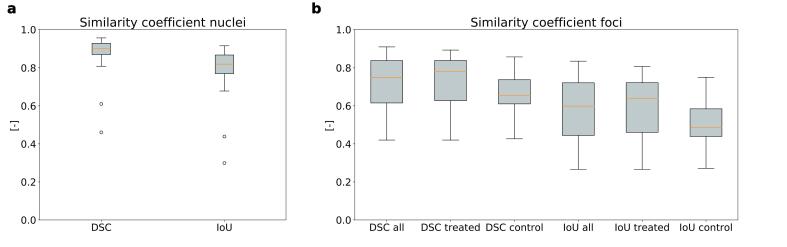
Boxplots of the pixel wise Dice similarity score (DSC_pixel_) and intersection over union (IoU_pixel_) on the test set comparing the semantic model predictions to the ground truths. Grey lines represent the median, the boxes the 25^th^ to 75^th^ percentiles. Note that the error bars represent the 5^th^ to 95^th^ percentile. a): the similarity coefficients of the nuclei on the test set. The DSC_pixel_ on the left and the IoU_pixel_ on the right. b): the similarity coefficients of the foci on the test set. From left to right: the DSC_pixel_ on the total test set, the DSC_pixel_ on the samples of the test set treated with photon radiation, the DSC_pixel_ on the samples of the test set kept in control conditions, the IoU_pixel_ on the total test set, the IoU_pixel_ on the samples of the test set treated with photon radiation, and the IoU_pixel_ on the samples of the test set kept in control conditions.

**Fig. 3:**
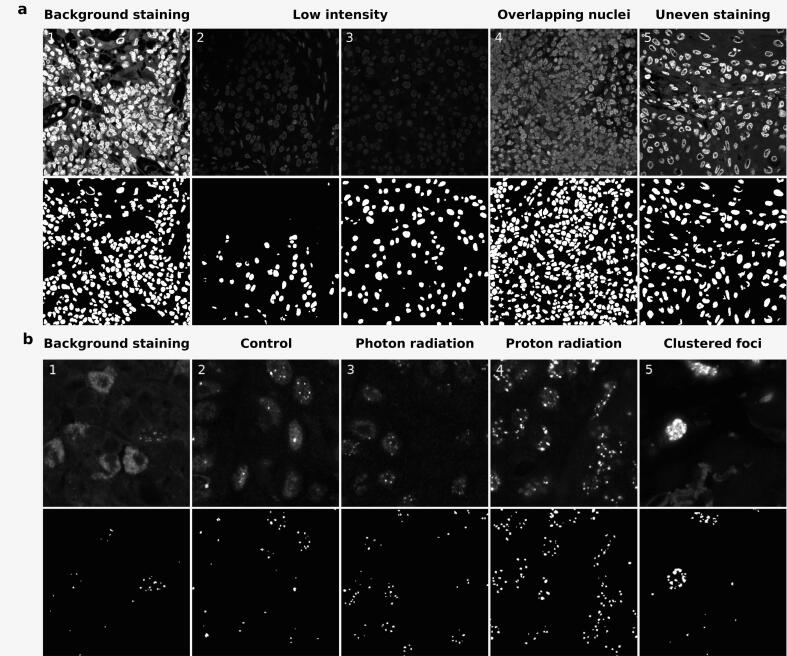
Example images and segmentation of the nuclei (DAPI) and foci (53BP1) images with different appearances from the test set. a): DAPI signal (top row) and corresponding semantic segmentations (bottom row). 1. An example with high background staining (DSC_pixel_ = 0.889). 2. An example with a low signal and the lowest DSC of the test set (DSC_pixel_ = 0.460). 3. an example with a low signal and higher DSC (DSC_pixel_ = 0.808). 4. An example of many overlapping nuclei (DSC_pixel_ = 0.870). 4. An example of decreased DAPI signal in the center of nuclei (DSC_pixel_ = 0.926).b): Images of 53BP1 foci (top row), and corresponding semantic segmentations (bottom row). 1. an example with high background staining (DSC_pixel_ = 0.420). 2. An example of the control condition(DSC_pixel_ = 0.726). 3. An example irradiated with photons (DSC_pixel_ = 0.839). 4. An example irradiated with protons (DSC_pixel_ = 0.910). 5. An example of clustered foci (DSC_pixel_ = 0.879).

Image analysis took ∼ 8 s per image (Supplementary Information 3). Semantic nuclei segmentations of the test set resulted in a median DSC_pixel_ and IoU_pixel_ of 0.90 (range: 0.46–0.96) and 0.82 (range:0.30–0.92), respectively (Fig. 2a). In most images, the nuclei could be segmented accurately even if their appearances in the images were very different (Fig. 3a). The exception were two outliers (8 %), which both had a very low staining intensity (Fig. 3a2). Note that other images with low staining intensity were segmented well (Fig. 3a3). For the validation set, the median DSC_pixel_ and IoU_pixel_ were comparable to the test set 0.90 and 0.82.

For the semantic 53BP1 foci segmentation, the median DSC_pixel_ and IoU_pixel_ were 0.75 (range: 0.42–0.91) and 0.60 (range: 0.27–0.84) on the total test set (Fig. 2b). Within the test set, the DSCs_pixel_ were slightly, but insignificantly, higher for samples treated with photons compared to corresponding control samples (DSC_pixel_ = 0.78 vs. 0.65 and p = 0.098) (Fig. 2b). Samples treated with protons had similar DSC_pixel_ as their counterparts treated with photons (0.88 and 0.84 respectively). Fig. 3b shows examples of different conditions and appearances of foci images. The DSC_object_ was 0.72 on the test set. On the validation set, the median DSC_pixel_ and IoU_pixel_ were 0.80 and 0.67, respectively.

**Fig. 4:**
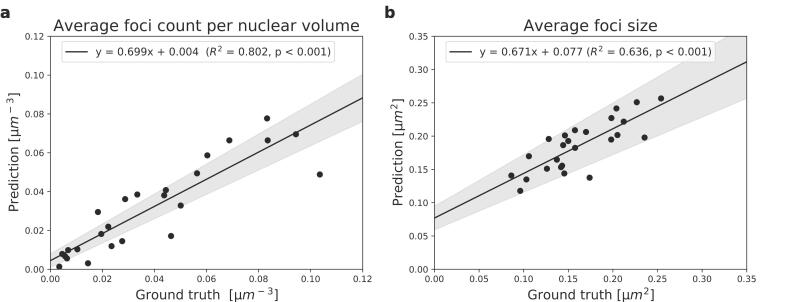
The correlation between the model predictions and ground truths on the test set. The dots reflect the average values per image and the lines represent linear regressions. The grey areas reflect the standard errors on the slopes and intercepts. a): Correlations in number of foci per µm^3^ nuclear volume (R^2^ = 0.802). b): Correlations in the size of the foci (R^2^ = 0.636).

An overlay of the foci and nuclei segmentations was created to compare predictions to the ground truth. Both the foci number per nuclear volume and foci size closely correlated between prediction and ground truth (R^2^ = 0.802 and 0.636, p < 0.0001 in both cases) (Fig. 4).

**Fig. 5:**
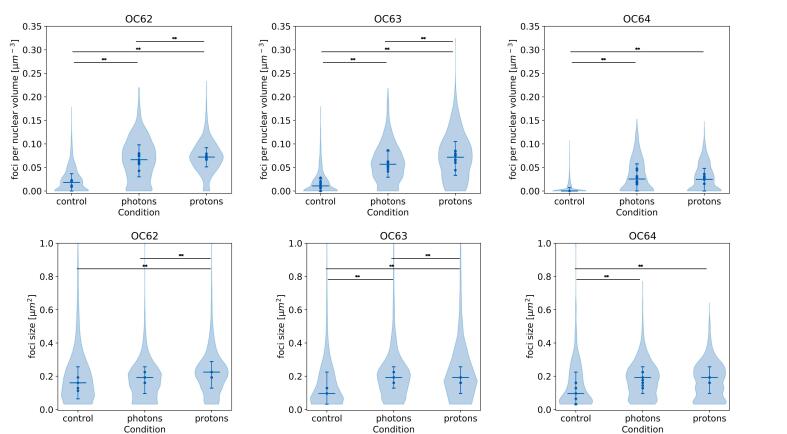
Violin plots depicting the foci count per nuclear volume (top row) and foci size (bottom row) in untreated OC samples, samples treated with either photon, or proton irradiation. The shape of the violin plot is determined by all individual nuclei in all images per condition. The dots are the median value per image, the horizontal lines represent the median, and the error bars indicate interquartile ranges of all nuclei.

Next, the model was applied to assess differences between photon, proton and no irradiation. For all patients, a significantly increased foci number was found for the treated compared to the control samples (Supplementary Information 2). For two out of three proton samples, the foci number and size was significantly higher than in the photon condition. (Fig. 3b2-4). Patient OC64 did not show a significant increase in foci number or size when irradiated with protons compared to photons. For this patient, a low foci number in all conditions was observed (Fig. 5). Even though each image contained on average hundreds of nuclei (rang 14–450 nuclei), large differences between images in the same condition were observed in number of foci per nuclear volume (average relative sd: 24.7 %) and average foci size (average relative sd: 11.2 % (Also see blue dots in Fig. 5).

## 4 Discussion

The DNA damage response after *ex vivo* irradiation of a tumor biopsy tissue could provide useful information about the radio-sensitivity of the individual tumor *in vivo* and may, therefore, be an important steps towards treatment personalization. However, manual evaluation of the DNA damage response would take hours per image and is subjective to inter-observer variability. Therefore, manual evaluation would be unsuitable for clinical decision making and automation is needed. In this study, we developed a method that automatically assesses the DNA damage response of HNSCC tumor tissue irradiated *ex vivo* by processing images of tumor nuclei and 53BP1 DNA damage foci with an excellent accuracy. This model reduced image-analysis time from multiple hours to minutes (∼8 s per image (Supplementary Information 3)) and it has been made publically available (github.com/Iris456/FociDetectionModel). Moreover, it eliminates inter-observer variability, while also offering additional information about foci size that cannot be achieved through mere counting. An average standard deviation in foci count of 25.6 % was observed between different images of the same tumor slice under identical conditions. This highlights the need of assessing multiple images per condition and slice, otherwise this intrinsic variation could influence ability to detect real inter-condition differences. Because this model allows the assessment of multiple images in a short time span, it enables analyzing multiple time points and dose levels to creating dose response curves, while accounting for the intrinsic variation in the tumor.

The model’s ability to accurately segment nuclei is evidenced by the high median IoU_pixel_ (0.82) and DSC_pixel_ (0.90) values observed across different staining intensities and tissue appearances (Fig. 3a). Two cases (8 %) had unacceptable image quality, which negatively affected the number of detected nuclei. However, in practice the microscope settings would have been adjusted to yield higher quality images.

The foci segmentation achieved a slightly lower median IoU_pixel_ (0.60) and DSC_pixel_ (0.75) than the nuclei segmentation due to the small size and unclear boundaries of DNA damage foci, which also leads to largely varying counts even among experts [20]. Comparing the foci U-net's performance to previously published semantic segmentation foci models is challenging because most prior studies focused on images of cell cultures, which are often of high quality and, therefore, easier to analyze than the clustered cells in tumor tissue in the present study. Despite the more challenging conditions of nuclei and foci counting in tumor tissue, our model had a good performance compared to models evaluated on cell cultures. Our model's DSC_object_ (0.72) was higher or comparable to most studies that focused on cell cultures. Vicar et al. reported median DSC_object_ values of 0.22, 0.38, 0.49, and 0.67 for FocAn, AutoFoci, CellProfiler, and DeepFoci, respectively [28]. Hohmann et al. developed machine learning models for foci detection in cell cultures, resulting in a median DSC_object_ between ∼ 0.64 and 0.80 [24]. The median DSC_object_ between observers manually counting foci in cell cultures (0.75) was similar to our model's performance on tissue cultures [20]. Although one machine-learning model based on cell cultures achieved a DSC_object_ higher than 0.9 [20], its retraining on our training set with tumor tissue led to a considerable lower DSC_object_ of only 0.66 on our training set, in contrast to 0.81 achieved on the training set by our current model (see Supplementary Information 4).

The correlation of predicted foci per nuclear volume with the ground truth was higher for the current model in tissue cultures (R^2^ = 0.801), than of previous models for cell cultures (FocAn: R^2^ = 0.04; AutoFoci: R^2^ = 0.01; CellProfiler: R^2^ = 0.01; DeepFoci: R^2^ = 0.75), and inter-observer correlation (R^2^ = 0.60) on cell cultures presented by Vicar et al. (2021) [28]. Furthermore, strong correlations were observed between predictions and ground truths of foci sizes (R^2^ = 0.636).

To demonstrate feasibility of using the model to compare differences in DNA damage occurring in different conditions, the model was applied to *ex vivo* tissue cultures treated with protons, photons, or left untreated. In two of three patients, a significant increase in the number and size of foci was observed two hours after proton radiation compared to photon radiation, indicating more persisting and complex DNA damage after proton radiation. Even though the sample size is too small to draw definite conclusions, this is in line with the observation that proton radiation may lead to more complex DNA damage that cannot be repaired as efficiently as photon-induced DNA damage [19], [29].

Interestingly, differences in foci count between different regions in the tumor for the same conditions (the individual dots in Fig. 5) were on occasion larger than the average differences between treatment groups. Therefore, it is crucial to assess multiple images for the same condition to ensure correct conclusions about radio-sensitivity are being drawn. This is in agreement with the findings of Rassamegevanon et al (2017) [21]. The presented model made it feasible to assess multiple images in a reasonable amount of time (8 s/image), while with manual counting processing multiple images would be highly impractical due to counting times of hours per sample.

This study had a few limitations. First, the model was trained, tested, and validated on OCSCC tissue. OCSCC patients do not often receive primary radiotherapy. However, other HPV-negative HNSCC tumors *e.g.* oropharyngeal SCC (OPSCC) that do receive primary radiotherapy, are very similar to OCSCC in terms of pathology. Indeed, ongoing internal research shows that the model also works for OPSCC tumor biopsy tissue. Moreover, this research was focused on DAPI and 53BP1 stainings. A disadvantage of DAPI is that it also stains not cancerous nuclei and 53BP1 does not include all DNA damage. However, ongoing unpublished research shows that this segmentation tool can also be used for p63, which is a HNSCC tumor nuclei specific staining. Moreover, we expect that the foci counting would work for other DNA damage proteins, such as RAD51 foci. Third, the model was trained on one microscope with a constant objective and pixel size. When using the model on different microscopes and settings, the performance of the model should be verified.

In conclusion, we created, validated, and tested an accurate model that enables reliable and rapid *ex vivo* radio-sensitivity assessment in terms of the amount of unrepaired DNA damage and complexity of DNA damage. The model is made publicly available (github.com/Iris456/FociDetectionModel). This enables, for the first time, comprehensive analyses of DNA damage in *ex vivo* tissue cultures within minutes and therefore within the time frame for clinical decision making. Therefore, it opens the door to treatment personalization for HNSCC based on *ex vivo* response of tumor tissue acquired through biopsies as biomarker.

## CRediT authorship contribution statement

**I. Lauwers:** Conceptualization, Methodology, Software, Validation, Formal analysis, Investigation, Data curation, Writing – original draft, Visualization, Project administration. **K.S. Pachler:** Methodology, Investigation, Writing – review & editing. **M.E. Capala:** Conceptualization, Methodology, Investigation, Writing – review & editing, Supervision, Project administration. **N.D. Sijtsema:** Methodology, Writing – review & editing. **D.C. Van Gent:** Methodology, Writing – review & editing, Funding acquisition. **M. Rovituso:** Resources, Writing – review & editing. **M.S. Hoogeman:** Methodology, Writing – review & editing, Supervision. **G.M. Verduijn:** Conceptualization, Methodology, Writing – review & editing, Supervision, Project administration. **S.F. Petit:** Conceptualization, Methodology, Writing – review & editing, Supervision, Project administration, Funding acquisition.

## Declaration of competing interest

The authors declare the following financial interests/personal relationships which may be considered as potential competing interests: This study was funded by a research grant of The Dutch Cancer Society (KWF 2019-12141). The Erasmus MC Cancer Institute also has research collaborations with Varian, a Siemens Healthineers Company, Elekta AB, Stockholm, Sweden, and Accuray Inc, Sunnyvale, USA.
