# Supplementary material for: Ex vivo radiation sensitivity assessment for individual head and neck cancer patients using deep learning-based automated nuclei and DNA damage foci detection
Source: Clin Transl Radiat Oncol. 2024 Jan 30;45:100735. doi: 10.1016/j.ctro.2024.100735 (PMC10877102; doi:10.1016/j.ctro.2024.100735)
Supplement: Supplementary Data 1 [file mmc1.docx]

# Supplementary information:

**Supplementary information 1: Ground truth generation foci**

To increase the efficiency of creating the ground truths of the foci, a semi-automatic method was used. The trainable Weka Segmentation plugin in ImageJ was applied using the standard settings. In the images, several pixels were manually classified as foci or background. Subsequently, the FastRandomForest algorithm assigned all pixels to either the background or foci class based on the manual classification. This segmentation was manually checked and incorrect pixels were manually reclassified. The algorithm then used the new information to improve the classification. This process was repeated for each image individually until the segmentation was accurate. The information obtained from earlier images was used to improve the FastRandomForest prediction in subsequent images. However, still manual adaption and retraining was necessary for each image individually to obtain an accurate segmentation. Note that due to the training and manual modification that is required per image individually, this method is not a viable alternative for the automated segmentation method proposed in the study. It was useful however to increase the efficiency of the process to generate the ground truth.

**Supplementary information 2: Applying the model to count the number of foci per nucleus in different conditions**

The model is applied to ten images per condition of both the treated and untreated sample of each sample in the test set. There was always a significant difference between the treated and untreated sample (p<0.05). The number and the foci per nuclear volume and the foci size are depicted in Figures S2A and S2B respectively.

**Supplementary information 3: The time required to perform the segmentation**

The analysis encompasses different steps. When segmenting 44 images in a batch processing setting, the required time in minutes was:

- Slice selection: 03:45 min
- Prediction DAPI: 0:28 min
- Prediction foci: 01:18 min
- Combine predictions: 0:05 min
- Post processing and analysis combined predictions: 03:10 min
- Total time: 05:46 min
- Time per image: ~8 seconds per image

**Supplementary information 4: FindFoci**

The FindFoci plugin ImageJ was trained on our training set. This application uses the coordinates of the foci in the ground truth to train the machine learning model. These coordinates were obtained by calculating the centers of the foci in the ground truths and storing them in an Excel file using python 3.8. Subsequently, this was used to train the FindFoci Optimiser Multiple-Image according to the manual [1].

This resulted in a DCE_object_ on the training set of 0.658. Moreover, the segmentations were not comparable to the ground truth nor the segmentation of our U-net model (Figure S4). Therefore, it could be concluded that FindFoci performed worse counting foci in tumor tissue compared to cell cultures.

[1] [FindFoci.pdf (sussex.ac.uk)](http://www.sussex.ac.uk/gdsc/intranet/pdfs/FindFoci.pdf)
