# Supplementary figures and images for: Ex vivo radiation sensitivity assessment for individual head and neck cancer patients using deep learning-based automated nuclei and DNA damage foci detection
Source: Clin Transl Radiat Oncol. 2024 Jan 30;45:100735. doi: 10.1016/j.ctro.2024.100735 (PMC10877102; doi:10.1016/j.ctro.2024.100735)

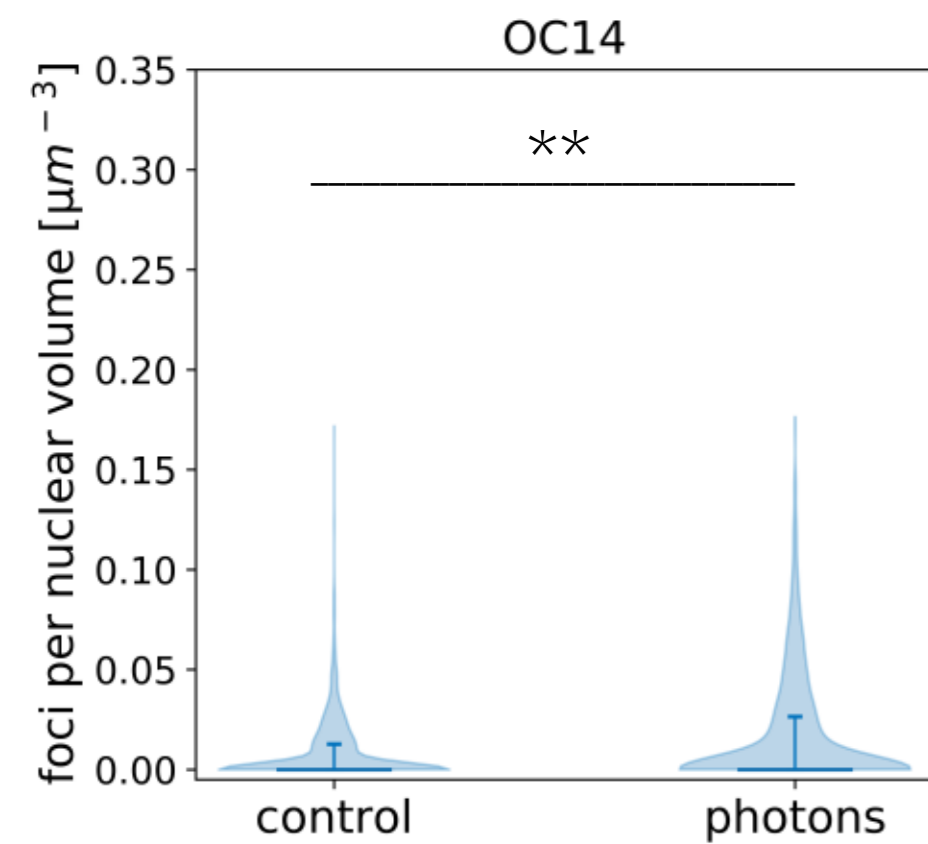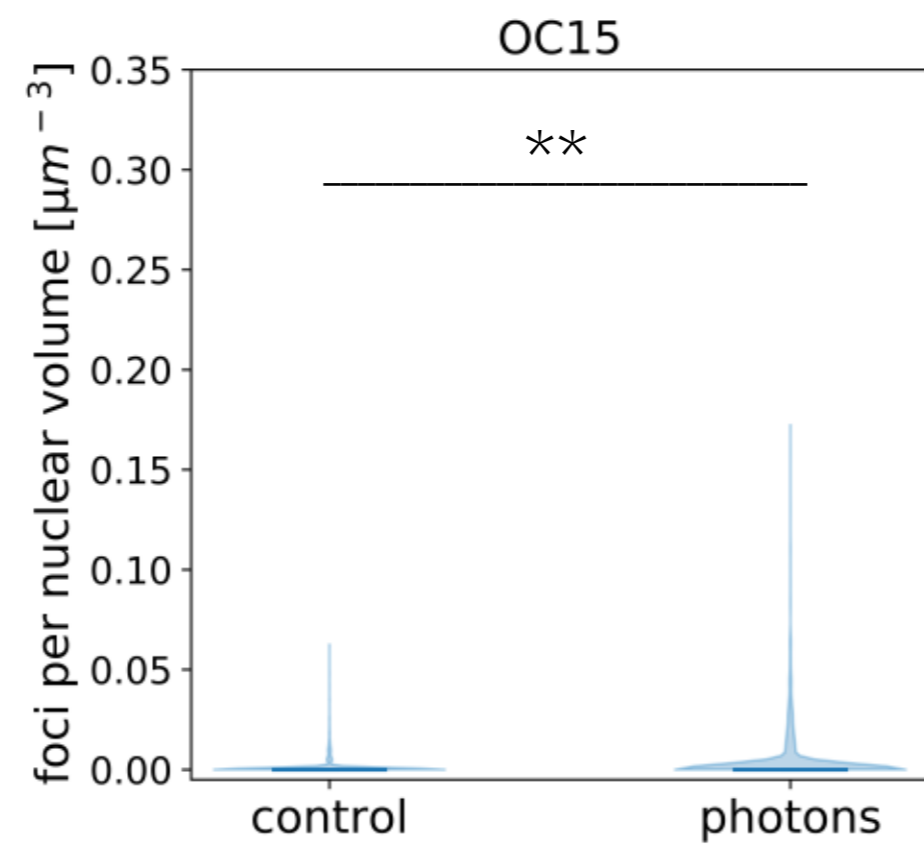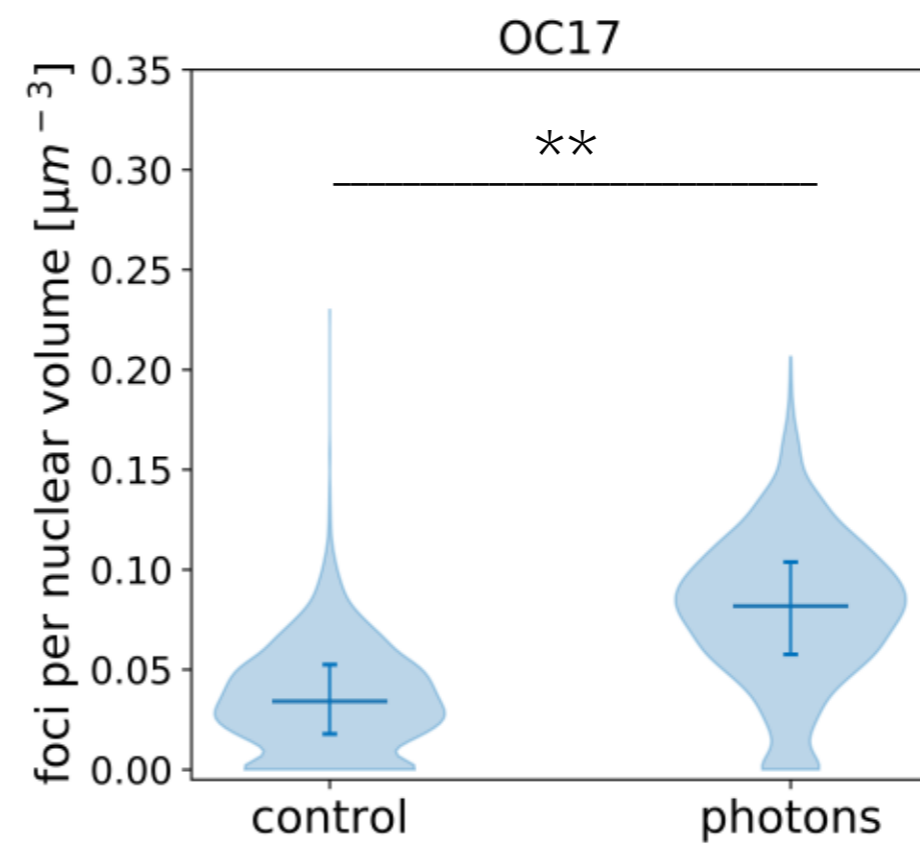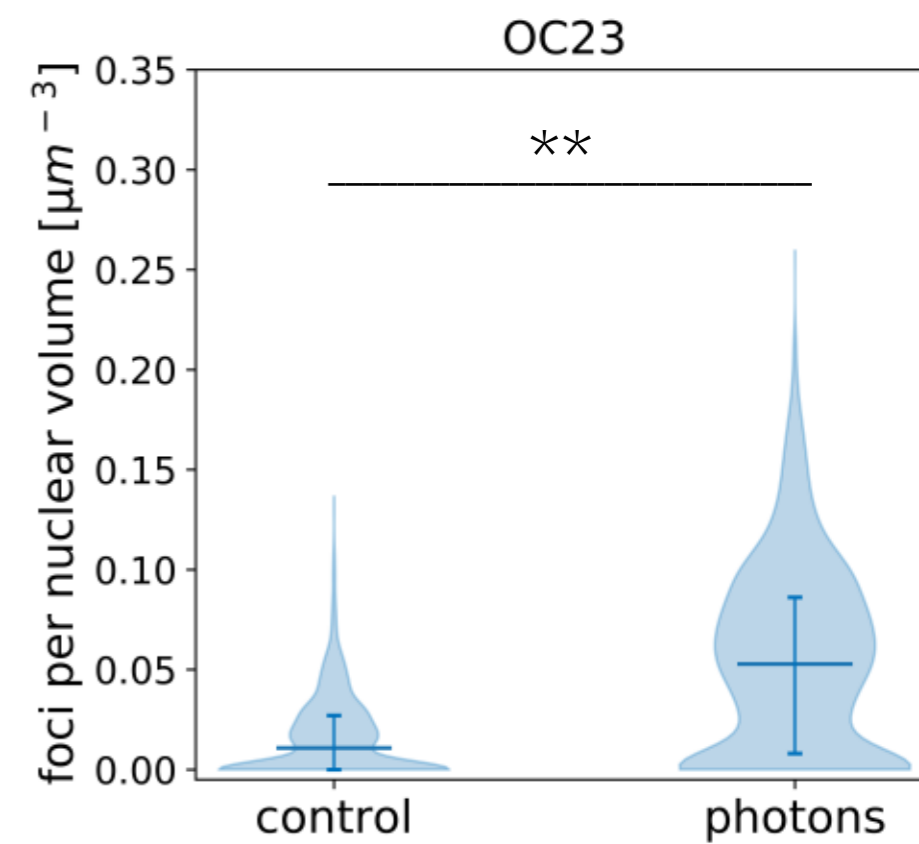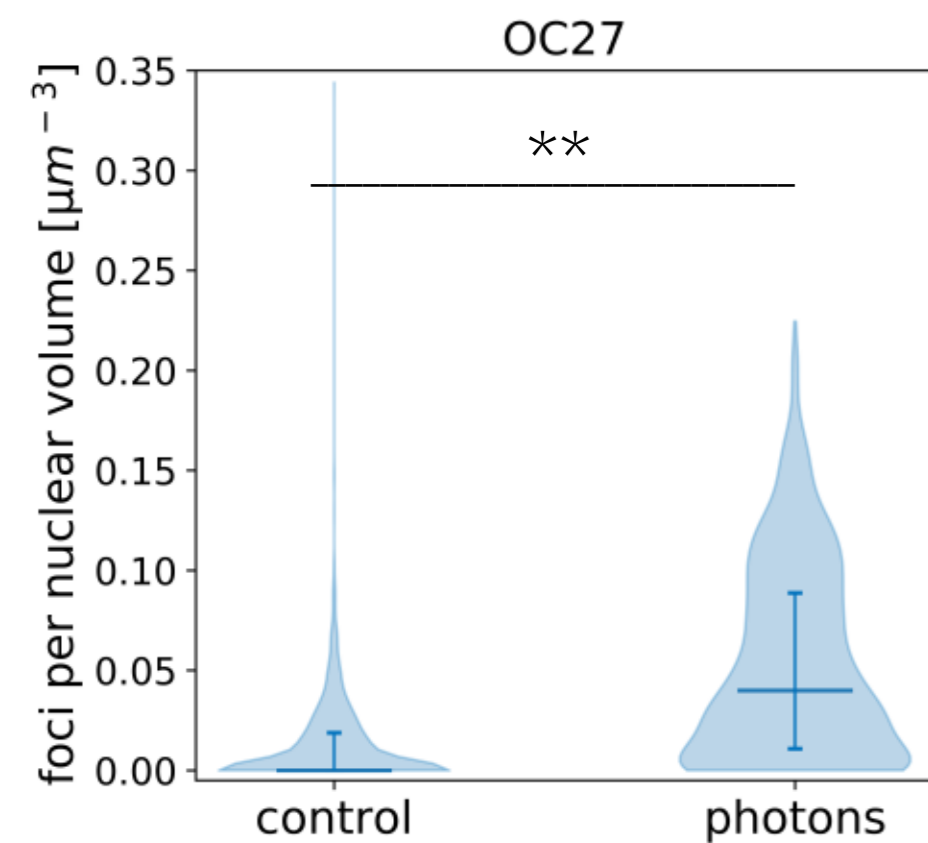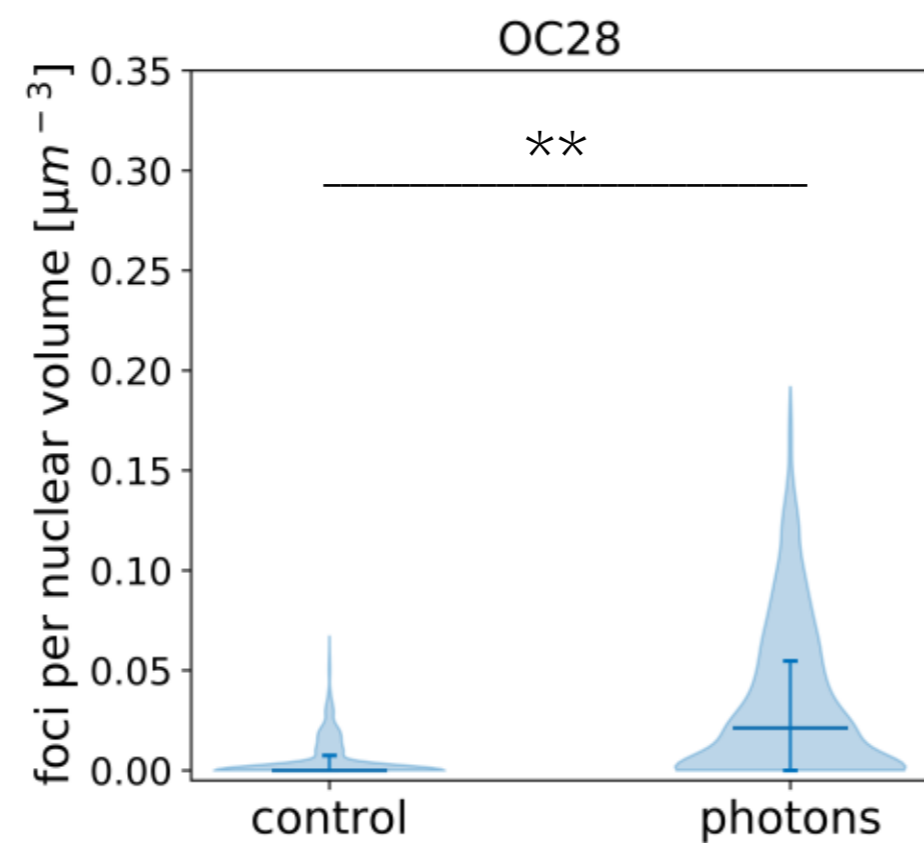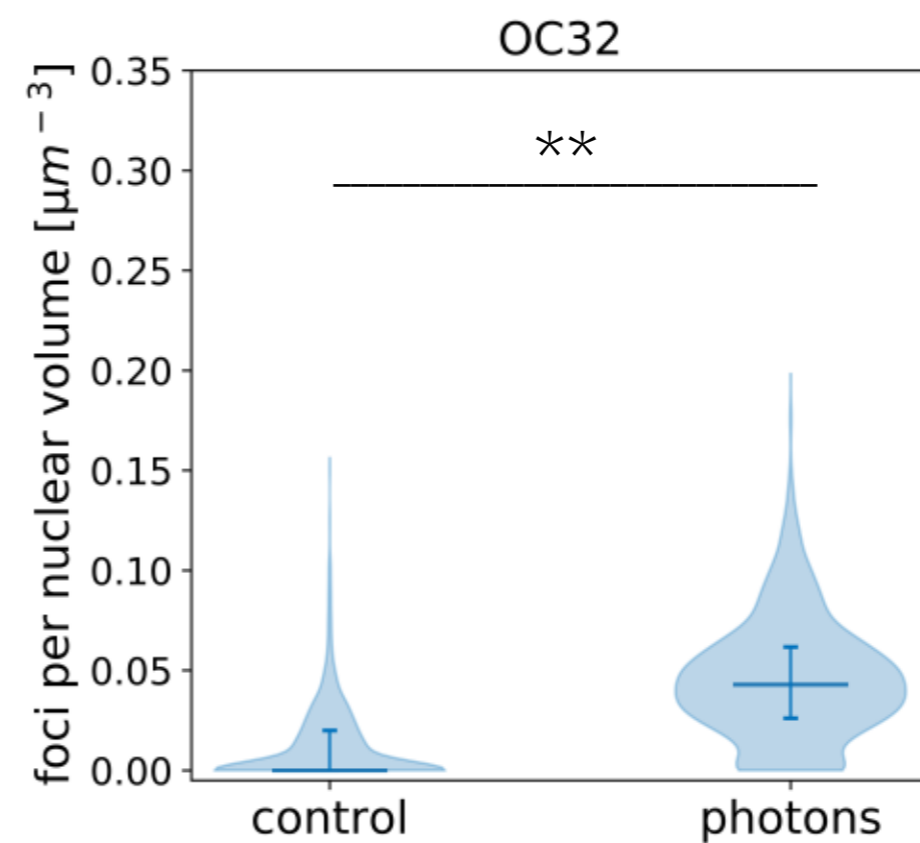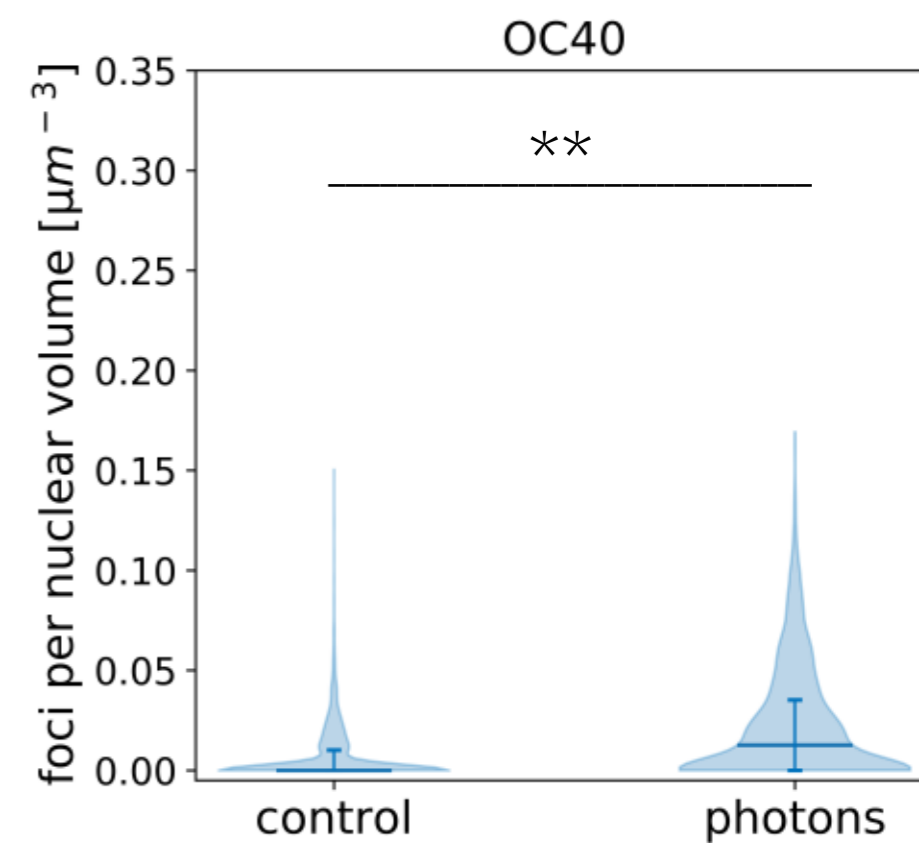

Supplement: Supplementary Data 2 [file mmc2.pdf]

OC14

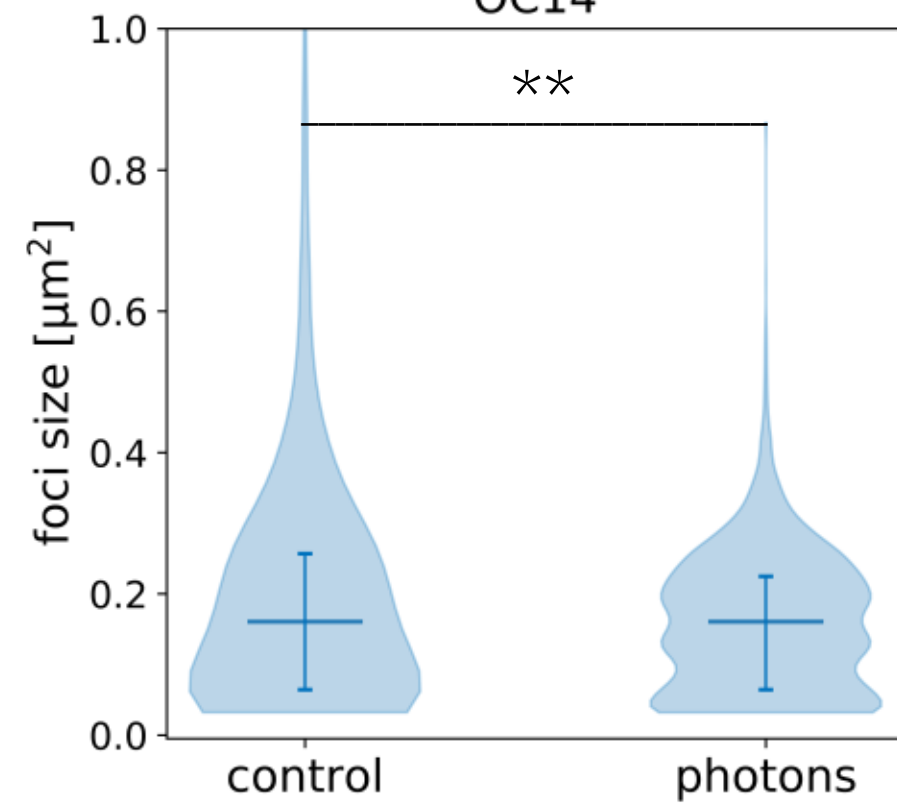

OC15

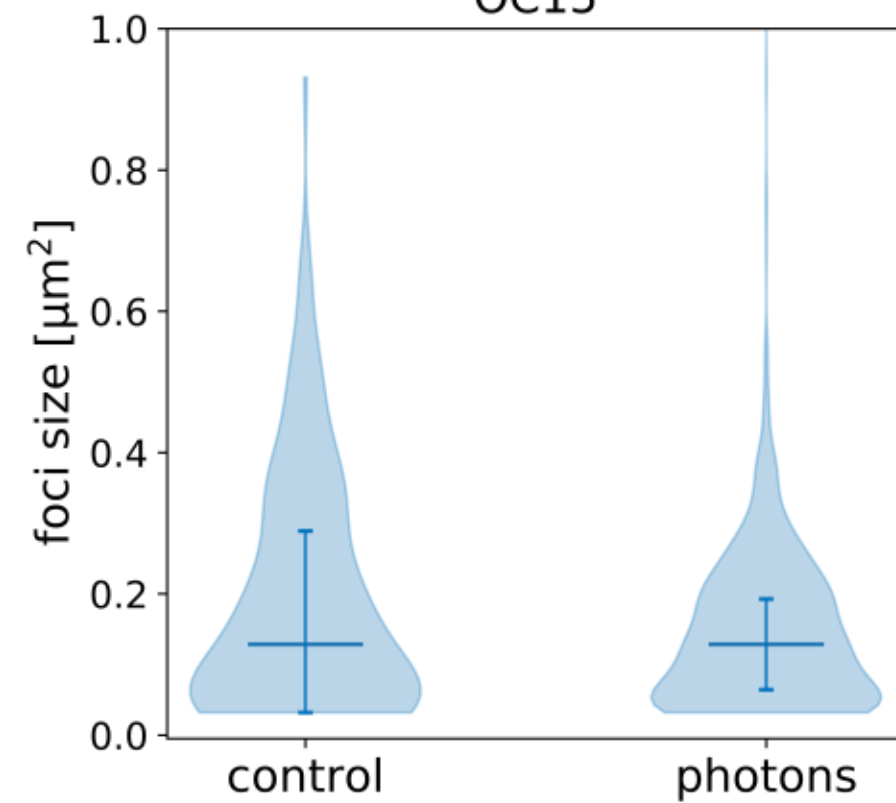

OC17

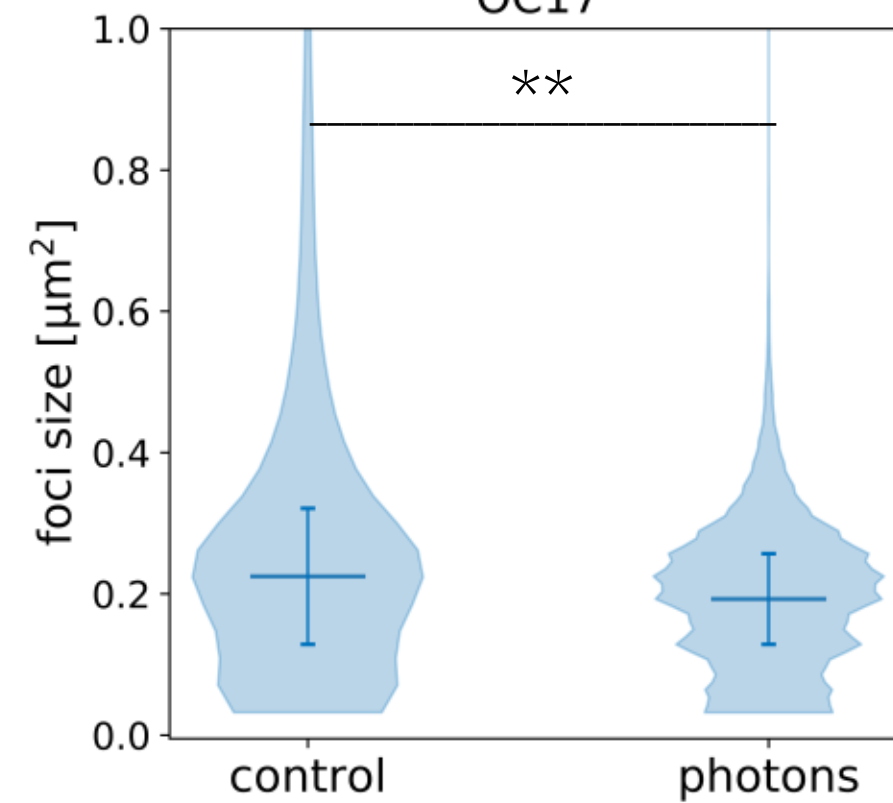

OC23

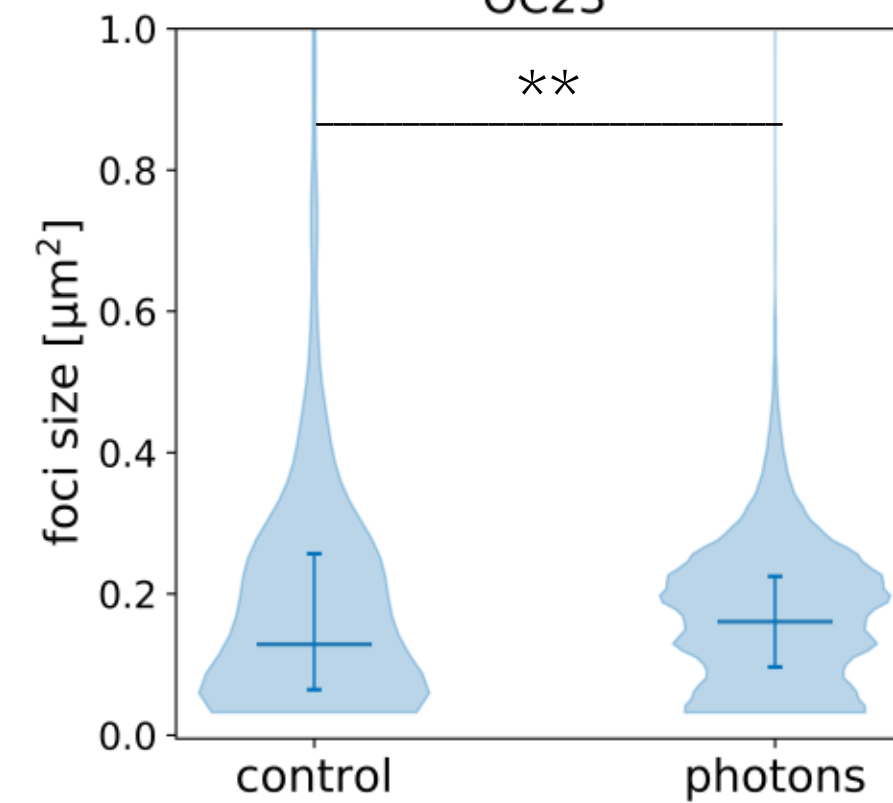

OC27

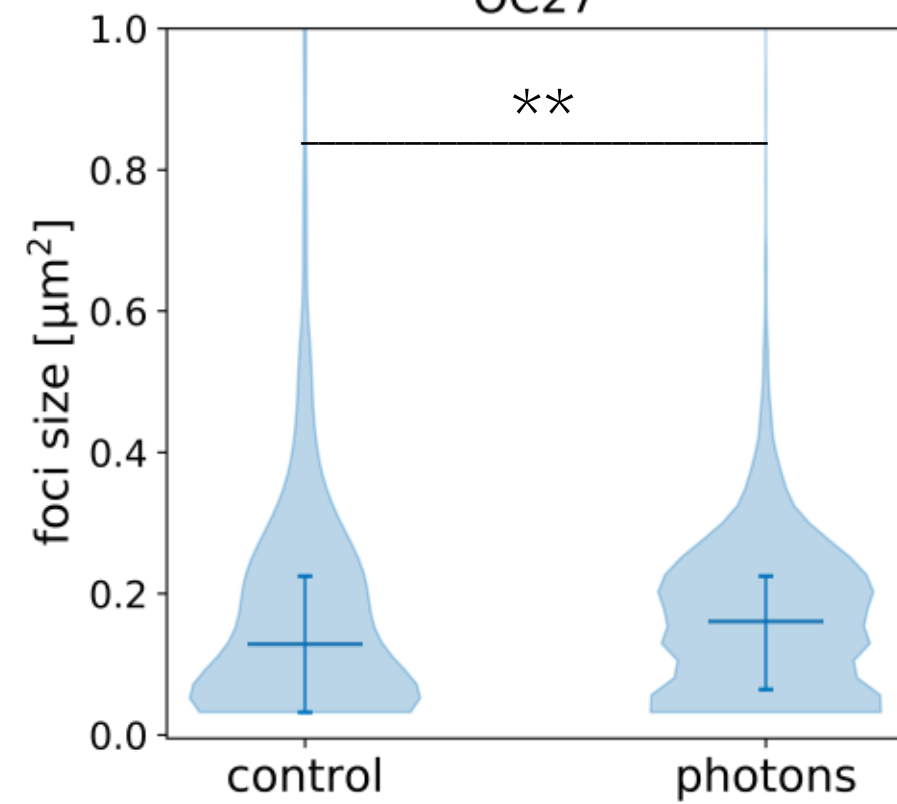

OC28

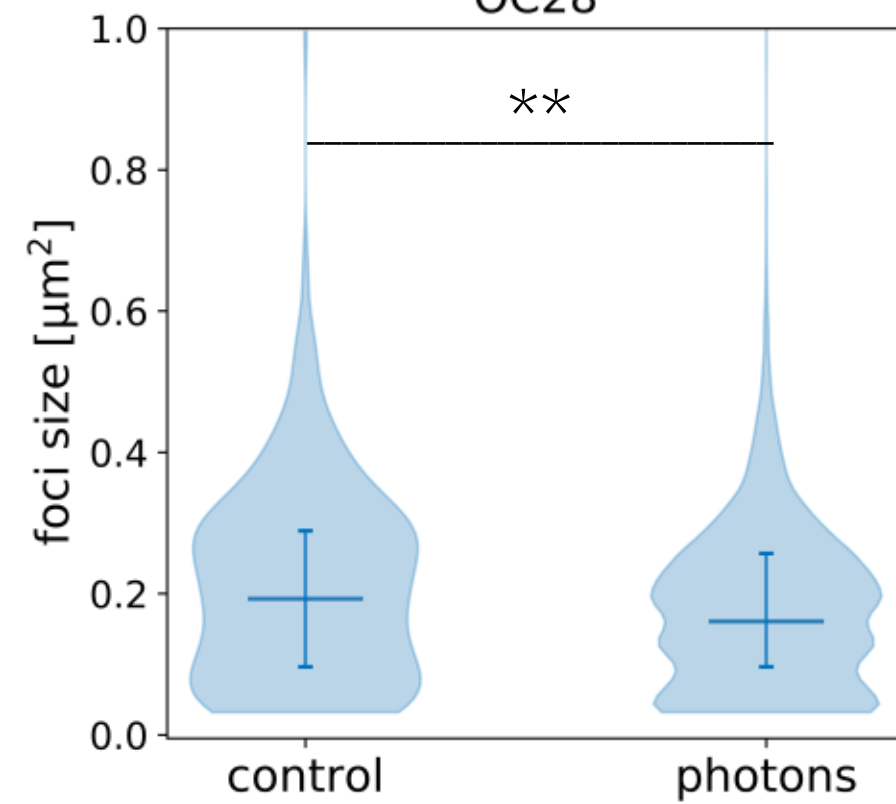

OC32

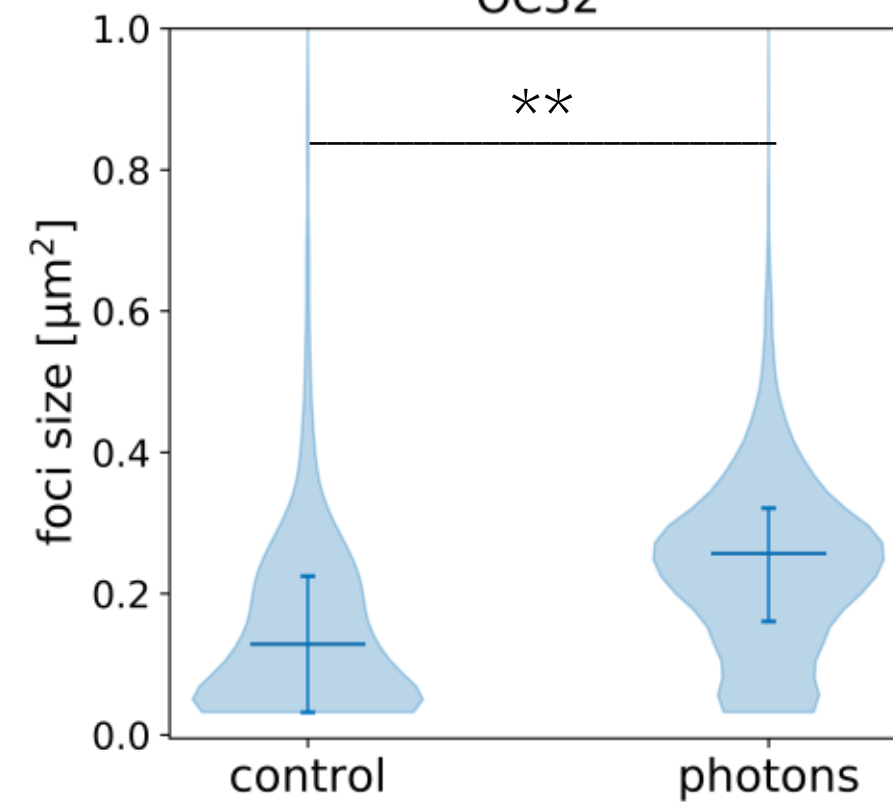

OC40

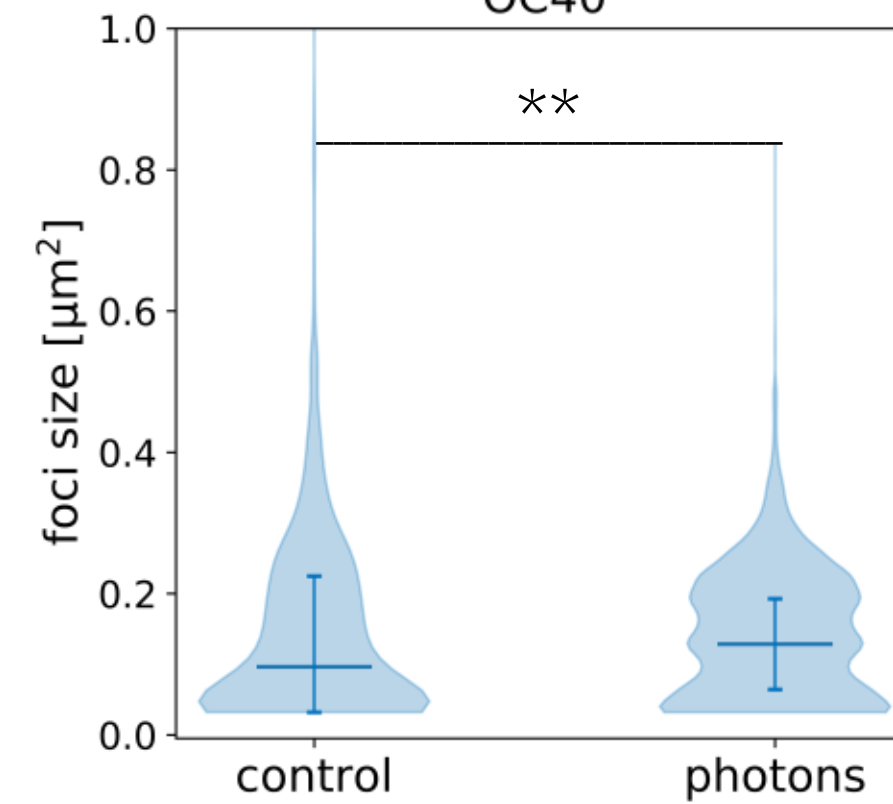

Supplement: Supplementary Data 3 [file mmc3.pdf]

Input

Ground truth

U-net

FindFoci

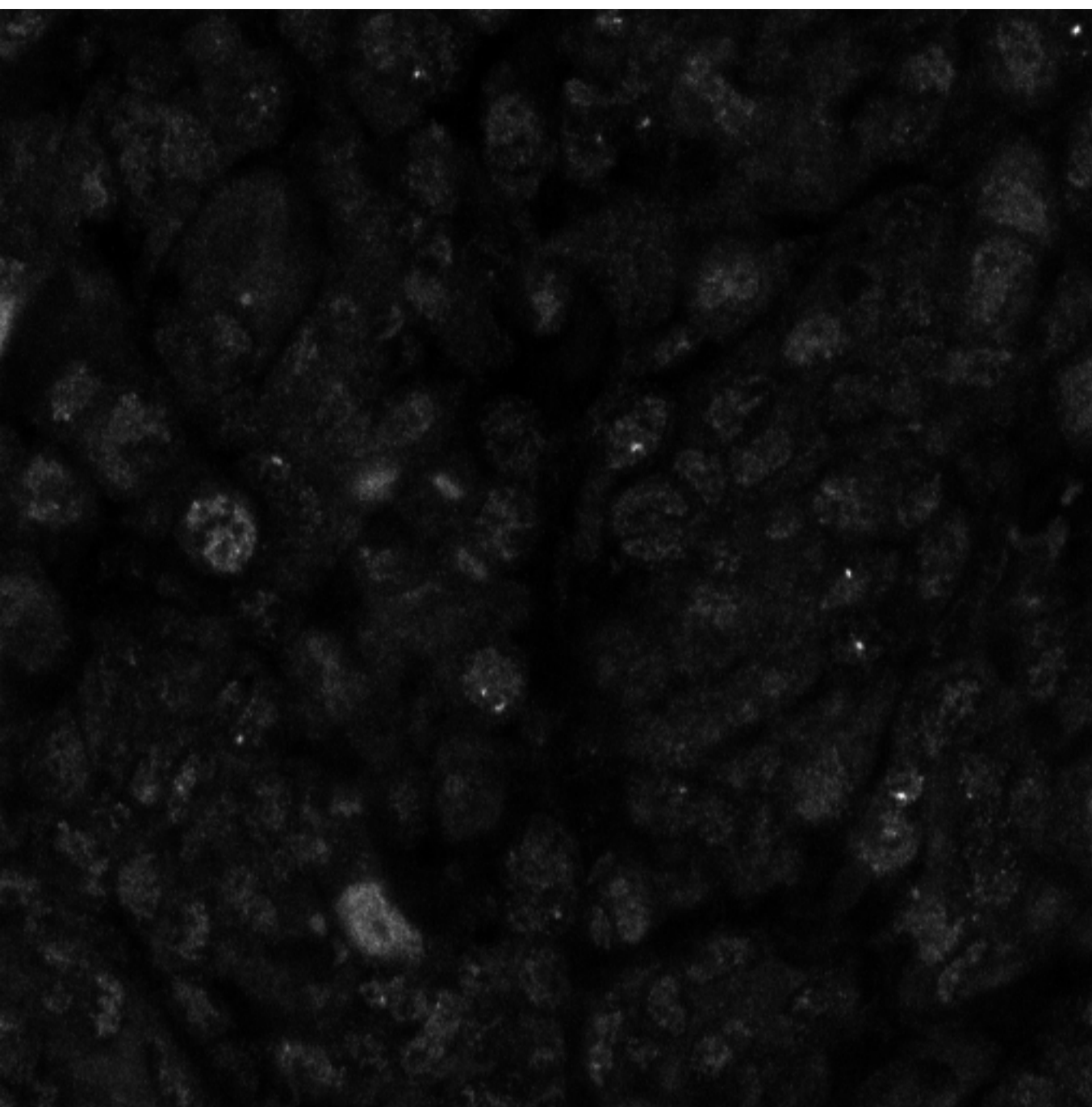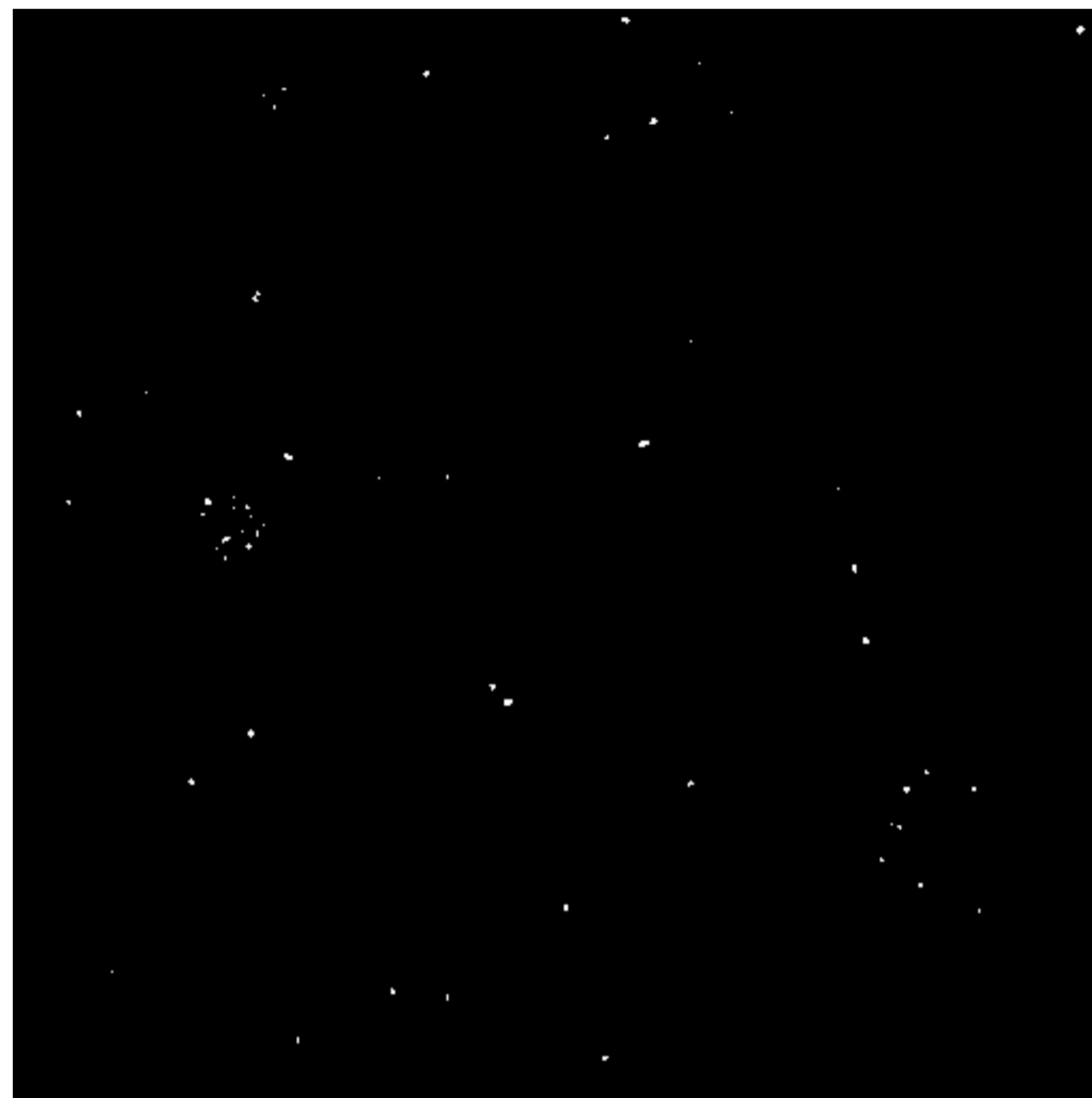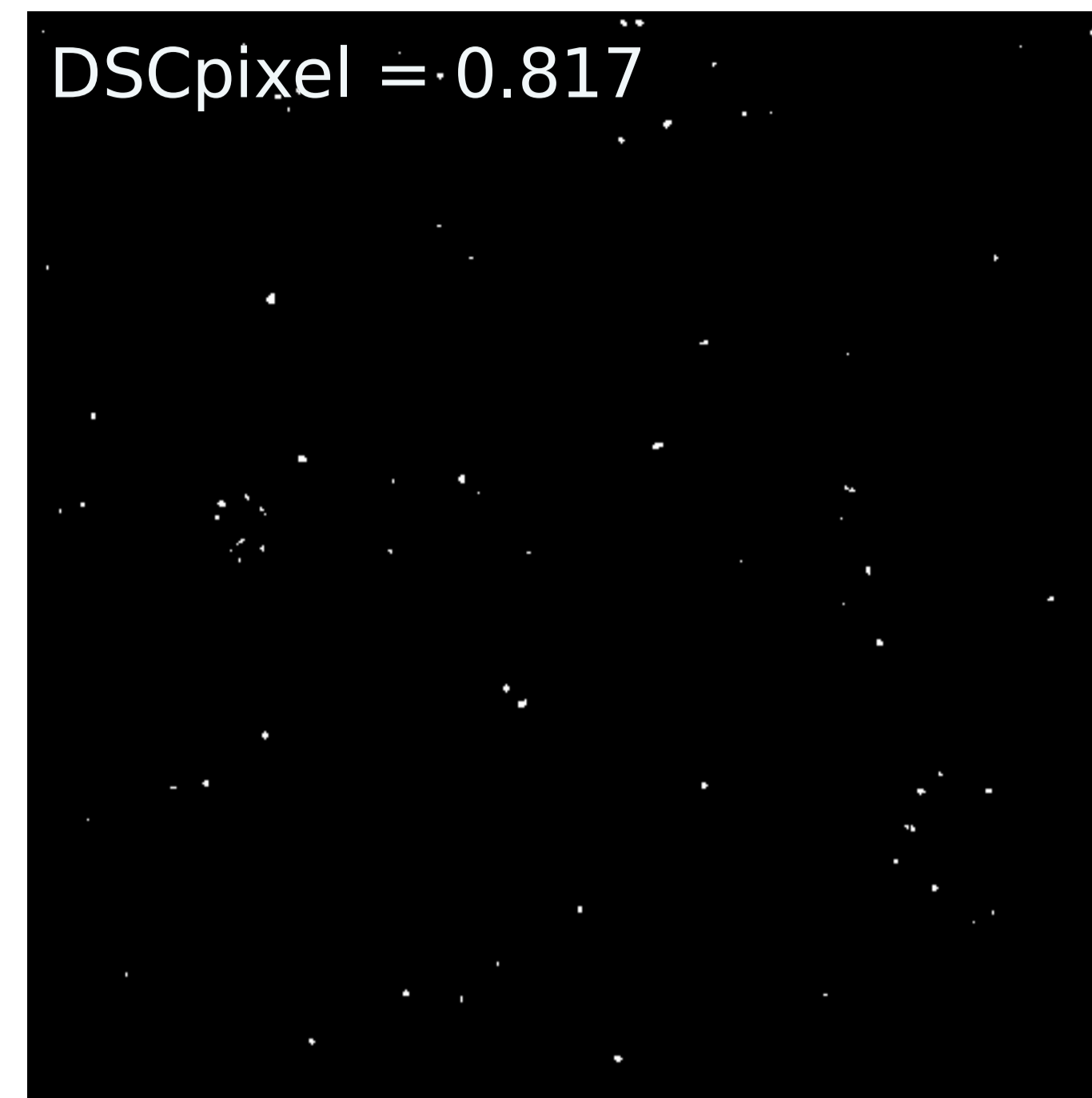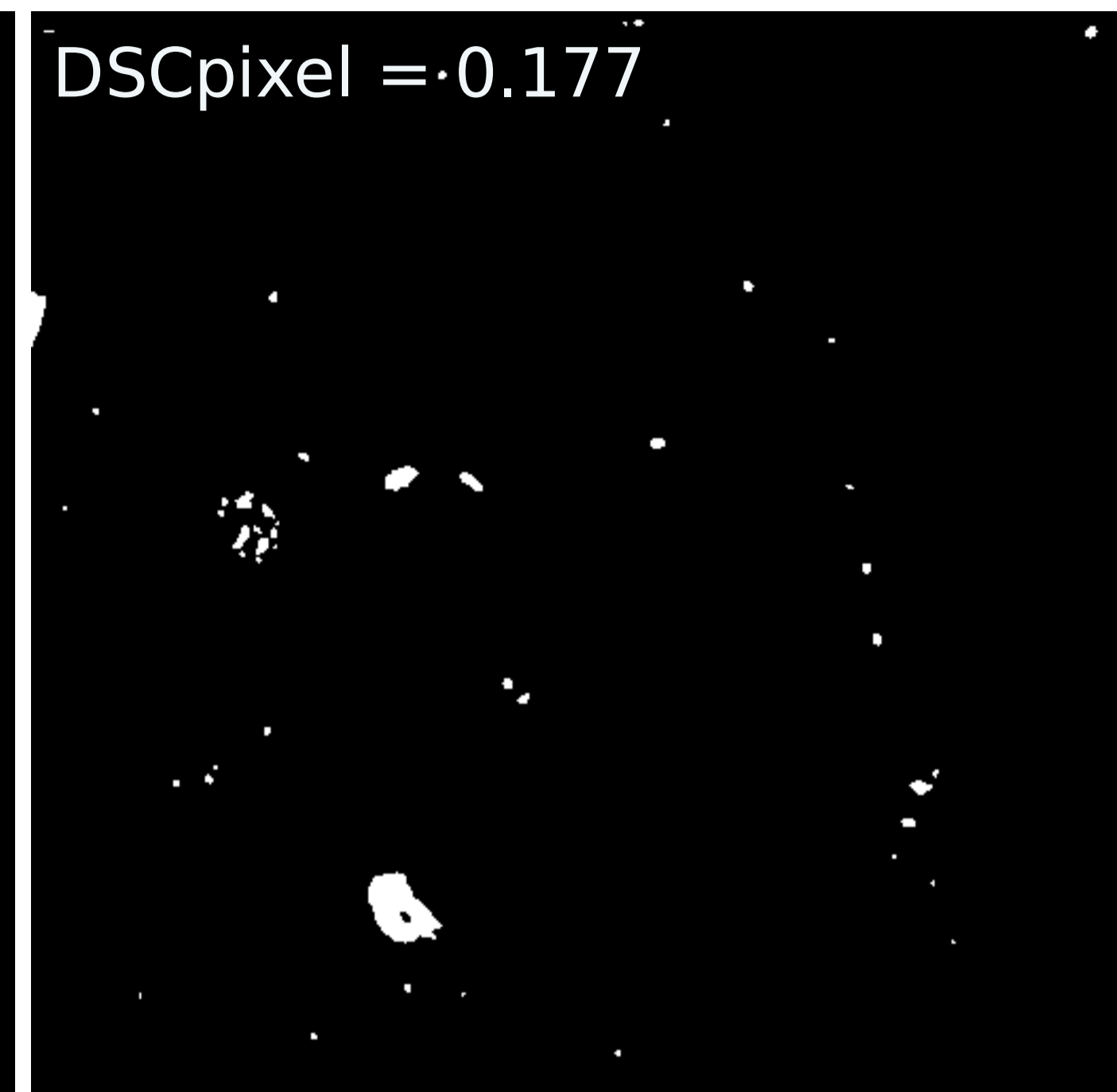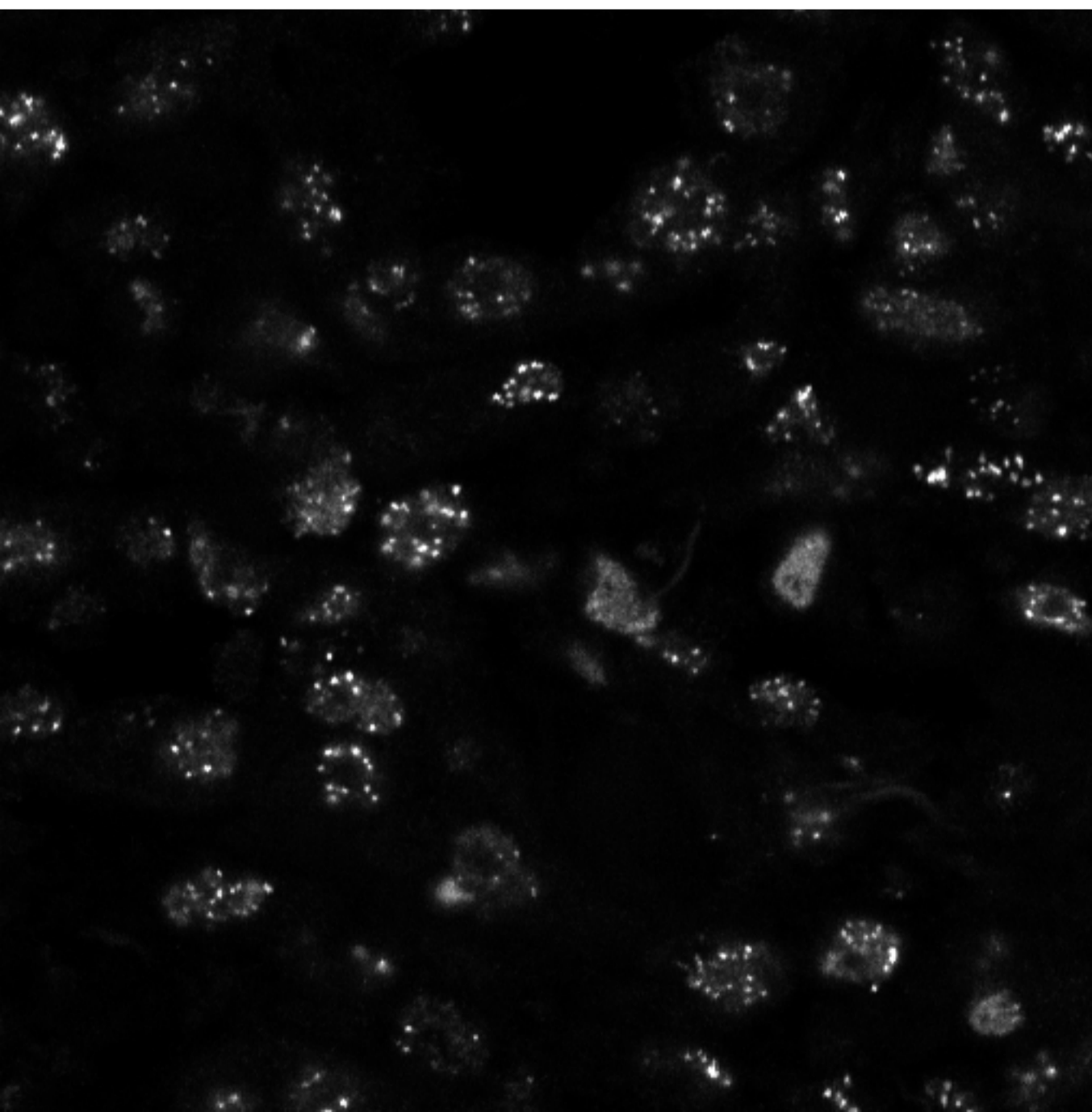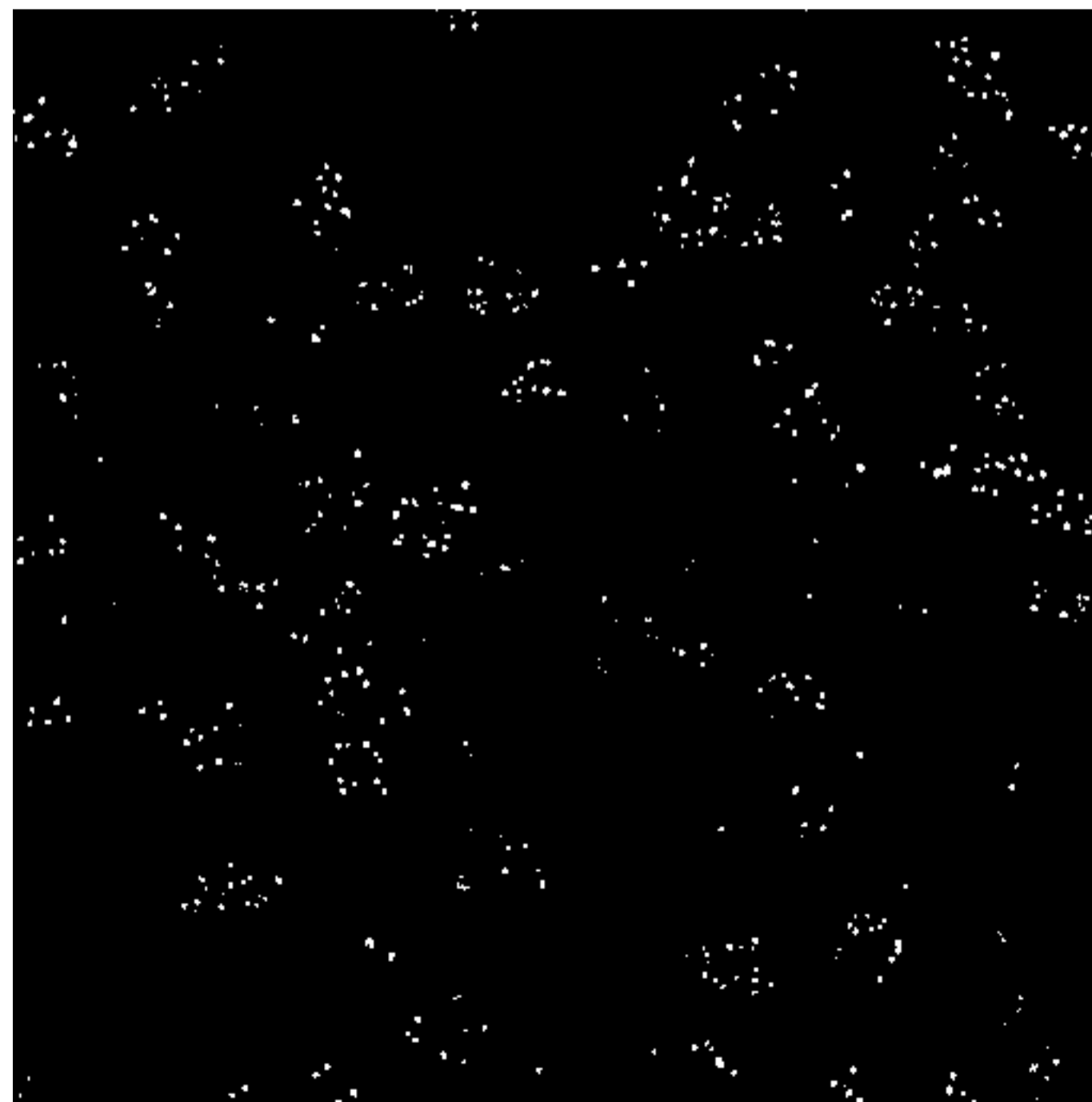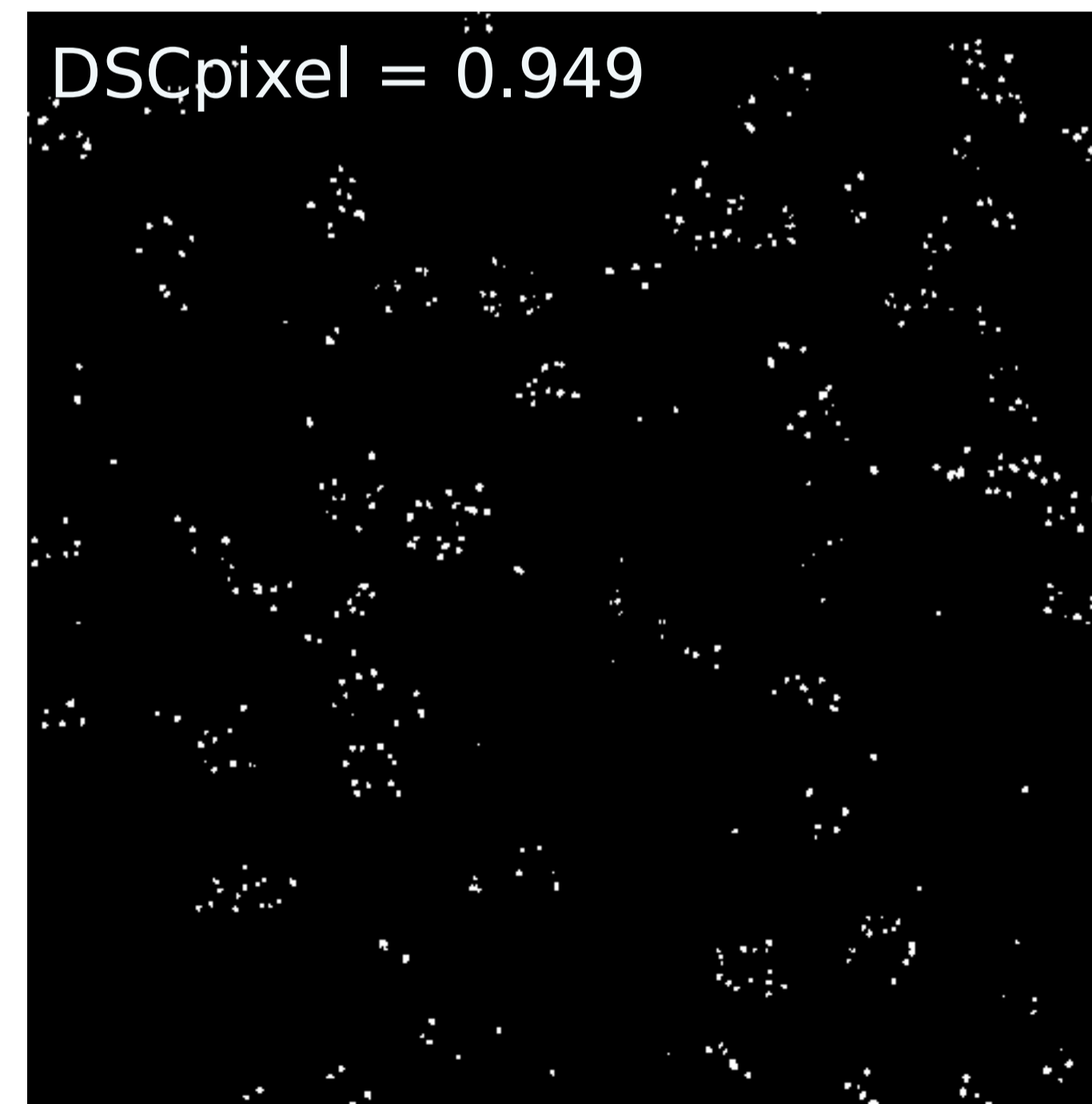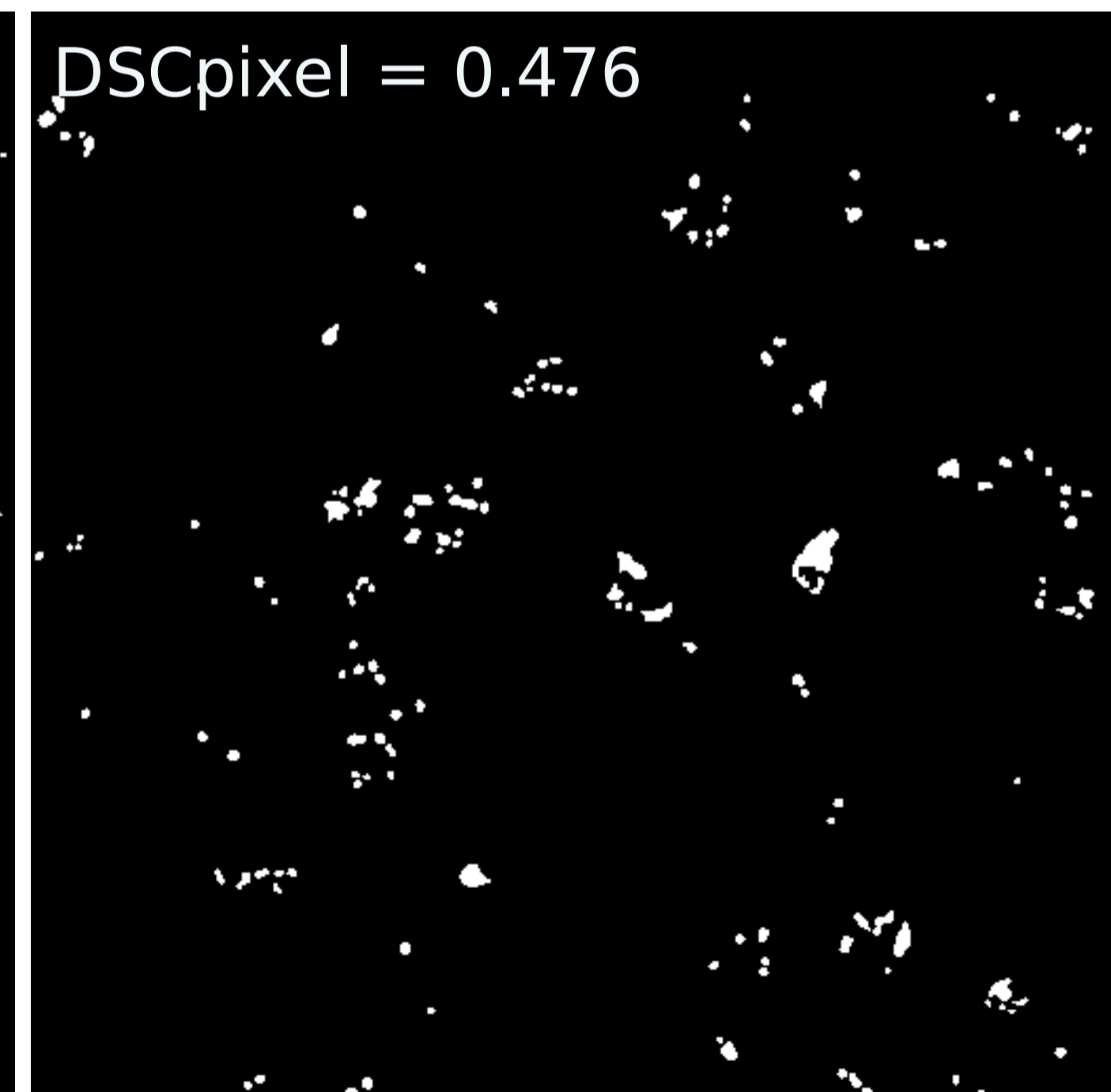

Supplement: Supplementary Data 4 [file mmc4.pdf]
